# Long-term single antiplatelet therapy for patients with acute coronary syndrome at high-risk for ischemia and bleeding: a precision cohort study

**DOI:** 10.3389/fcvm.2026.1798099

**Published:** 2026-05-01

**Authors:** Ma Wanda, Lei Mengjie, Wang Jingyao, Sun Xue, Wang Xiao, Li Cairong, Li Yachao, Zhao Zhigang, Xue Zengming

**Affiliations:** 1 Graduate School, Hebei North University, Zhangjiakou, China; 2 Langfang Core Laboratory of Precision Treatment of CAD, Langfang People’s Hospital, Langfang, China

**Keywords:** antiplatelet therapy, bleeding, high-risk, major adverse cardiovascular and cerebrovascular events, percutaneous coronary intervention

## Abstract

**Background:**

To compare the prognostic effects of different single antiplatelet therapy (SAPT) regimens (aspirin vs. clopidogrel) in patients with acute coronary syndrome (ACS) at high-risk for ischemia and bleeding following percutaneous coronary intervention (PCI) and switching from 12 months of dual antiplatelet therapy (DAPT) to SAPT.

**Methods:**

This study retrospectively analyzed 642 patients with ACS at high-risk of ischemia and bleeding who underwent PCI at a single-center between January 2017 and November 2022. After transitioning to SAPT, the cohort was divided into two groups based on the prescribed therapy: aspirin (*n* = 435) and clopidogrel (*n* = 207) groups. Patients were followed up for 24 months post-PCI. The primary endpoint was the occurrence of net adverse clinical events (NACE), including MACCE and major thrombolysis in myocardial infarction (TIMI) hemorrhage.

**Results:**

The incidence of NACE was comparable between the two patient groups. However, the clopidogrel group had a lower incidence of MACCE (4.3% vs. 9.2%, *P* = 0.031) and TVR (1.4% vs. 6.4%, *P* = 0.006) compared to the aspirin group. Cox regression analysis revealed that the incidence of MACCE and TVR in the clopidogrel group were 0.458 times [*hazard ratio [HR]*: 0.458; *95% confidence interval [CI]:* 0.222–0.944; *P* = 0.034] and 0.220 times (*HR:* 0.220; *95% CI:* 0.067–0.724; *P* = 0.013) that of the aspirin group, respectively.

**Conclusion:**

In patients with ACS at high-risk, transitioning to clopidogrel as SAPT after 12 months of DAPT and successful PCI was associated with a reduced incidence of MACCE and TVR compared to aspirin, without an increased risk of major bleeding events.

## Background

Acute coronary syndrome (ACS) encompasses a spectrum of conditions caused by coronary atherosclerotic plaque rupture or erosion, leading to complete or partial thrombosis. This includes unstable angina, ST segment elevation myocardial infarction (STEMI), and non-STEMI. Thrombosis, driven by platelet activation, adhesion, and thrombin generation, plays a central role in ACS pathophysiology. Consequently, intensified antiplatelet therapy is fundamental to its treatment. Guidelines recommend dual antiplatelet therapy (DAPT) for 12 months in patients with ACS, regardless of whether they undergo percutaneous coronary intervention (PCI) (1–3). Evidence from the PLATO and TRITON-TIMI 38 studies supports the combination of aspirin with potent P2Y_12_ receptor inhibitors, such as prasugrel or ticagrelor, as the preferred DAPT regimen for ACS management (2, 4). The duration of DAPT may be tailored based on individual ischemic and bleeding risks. For patients at high-risk for ischemia, extending the duration of DAPT can reduce adverse cardiovascular events, while shortening the therapy duration can lower bleeding risks in patients prone to bleeding. However, DAPT is not recommended for patients with high-risk for both ischemia and bleeding, as there is limited evidence on optimal strategies for balancing these risks. Current guidelines recommend transitioning patients with ACS to long-term oral aspirin monotherapy following 12 months of DAPT (5, 6). Although research has shown that compared to aspirin monotherapy, extending DAPT beyond 12 months can significantly reduce major adverse cardiovascular and cerebrovascular events (MACCE), it also increases the risk of bleeding (7–9). Therefore, prolonged DAPT exceeding 12 months may not be appropriate for patients with ACS at high-risk for both ischemia and bleeding. Currently, the optimal antiplatelet strategy for such patients remains unclear. The OPT-BIRISK study defined the “dual high-risk population” and randomly assigned patients who had completed 9–12 months of DAPT to clopidogrel monotherapy or a continued DAPT regimen of aspirin combined with clopidogrel for an additional 9 months. The study aimed to explore the prognostic outcomes between clopidogrel-based single antiplatelet therapy (SAPT) and aspirin plus clopidogrel DAPT in dual high-risk patients with ACS who underwent PCI (10). However, the study did not specify which SAPT regimen is most appropriate after 12 months of PCI. Building on the OPT-BIRISK study, our research focused on dual high-risk patients with ACS who underwent PCI. After completing 12 months of standard DAPT without experiencing MACCE or major bleeding events, patients were categorized into two groups based on their subsequent treatment regimen: one group received aspirin, and the other received clopidogrel as SAPT. The study then evaluated the 12-month prognostic differences between the two groups.

## Materials and methods

### Research participants

**Figure 1:**
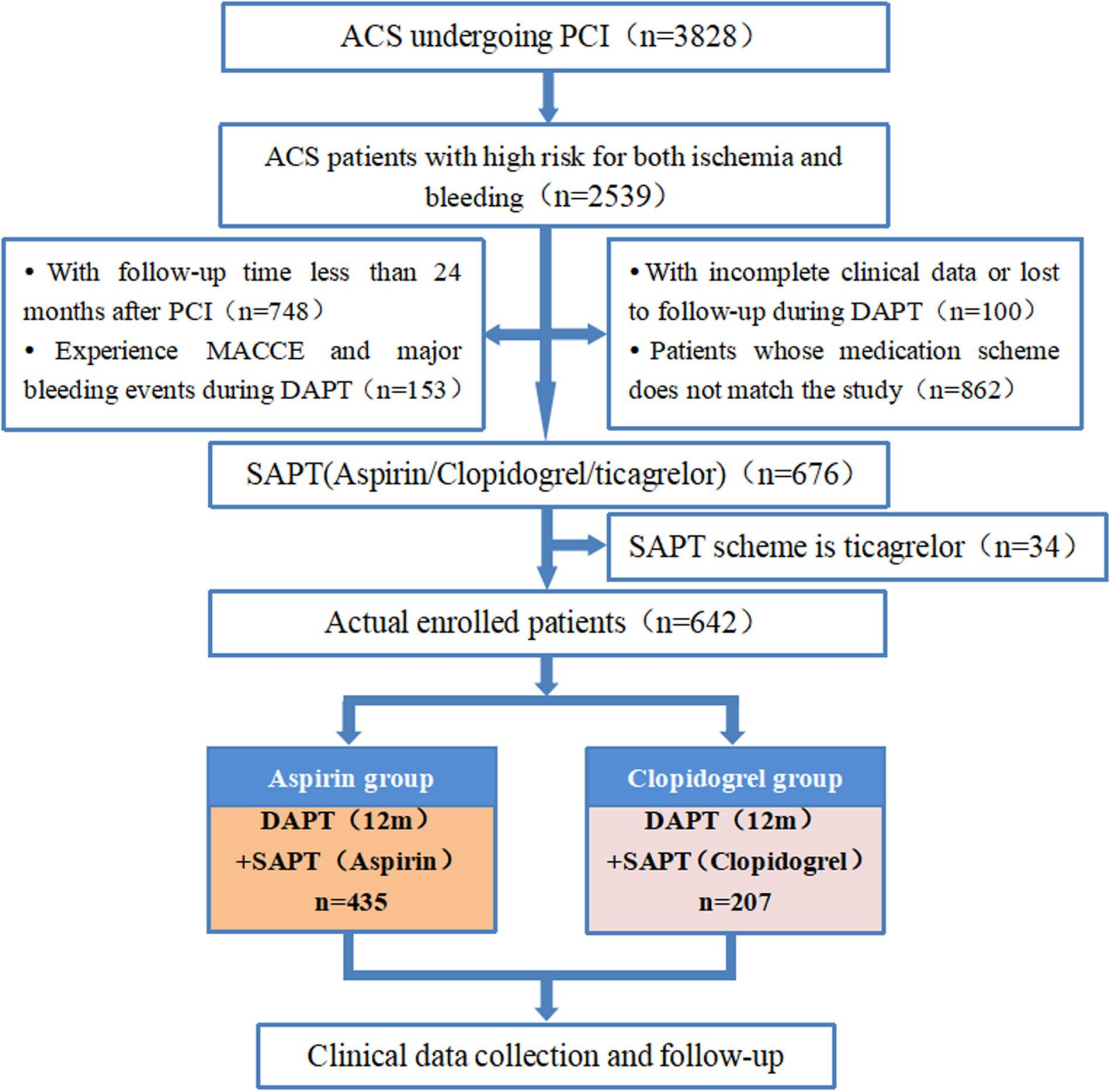
Flowchart for patient screening. ACS, acute coronary syndrome; PCI, percutaneous coronary intervention; MACCE, major adverse cardiovascular and cerebrovascular event; DAPT, dual antiplatelet therapy; SAPT, single antiplatelet therapy.

This single-center retrospective cohort study analyzed 676 patients with ACS at high-risk for both ischemia and bleeding who were hospitalized in the Department of Cardiology at Langfang People’s Hospital and underwent PCI between January 2017 and November 2022. All included patients had completed 12 months of DAPT without experiencing MACCE or major bleeding events during the therapy period. Following the transition to SAPT, they continued treatment for at least 12 months. Patients were categorized into three groups based on the SAPT type: 435, 207, and 34 cases in the aspirin, clopidogrel, and ticagrelor groups, respectively. Due to the small sample size and variability in ticagrelor dosing, the ticagrelor group was excluded, leaving the aspirin and clopidogrel groups for analysis. The patient screening process is outlined in Figure 1.

All participants included in this study met the relevant diagnostic criteria outlined in the European Society of Cardiology (ESC) guidelines (11, 12). The inclusion criteria were age ≥18 years, follow-up duration >24 months, typical symptoms of myocardial ischemia or infarction with supporting evidence from electrocardiograms or laboratory findings, and successful completion of PCI. Additionally, all patients completed the ischemia and bleeding risk assessment based on the OPT-BIRSIK criteria during hospitalization and were classified as dual high-risk patients with ACS (10). Patients who did not experience MACCE or major bleeding events during DAPT were included. The exclusion criteria were allergy or serious adverse reactions to aspirin or clopidogrel, inability to adhere to medication, severe hepatic insufficiency (Alanine aminotransferase or aspartate aminotransferase more than twice the upper limit of normal value), dialysis-dependent renal failure, and participation in other studies involving antiplatelet and anticoagulant therapies.

Dual high-risk: Patients aged <65 years were required to meet at least one clinical criterion for both high bleeding and ischemia risk defined in the OPT-BIRISK study; Patients aged 65–75 years were required to meet at least one clinical criterion for high bleeding or ischemia risk; Patients aged ≥75 years old were automatically considered dual high-risk (10).

A patient was considered at high ischemic risk if at least one of the following criteria was met in the OPT-BIRISK study: (1) Multivessel coronary disease, (2) Total stent length >30 mm, (3) Presence of thrombotic lesions, (4) Bifurcation lesions requiring double stent treatment (Medina classifications 0, 1, 1 or 1, 1, 1), (5) Lesions in the left anterior descending artery (≥50%) or proximal left anterior descending artery (≥70%), (6) Calcified lesions requiring rotational atherectomy, (7) ACS with positive troponin levels, (8) Diagnosed vascular disease, including previous myocardial infarction, ischemic stroke, peripheral artery disease (PAD), or coronary atherosclerotic heart disease (CAD)/PAD vascular reconstruction, (9) Recurrent myocardial infarction, coronary revascularization, stent thrombosis, or stroke occurring within 9 months prior to PCI, (10) Diabetes requiring medication (oral hypoglycemic therapy or subcutaneous insulin injection), and (11) Chronic kidney disease, defined as an estimated glomerular filtration rate (eGFR) < 60 mL/min/1.73 m^2^ or creatinine clearance rate <60 mL/min.

A patient was considered at high bleeding risk if at least one of the following criteria was met: (1) Female sex, (2) Iron deficiency anemia, (3) History of stroke (hemorrhagic or ischemic), (4) Diabetes requiring medication (oral hypoglycemic therapy or subcutaneous insulin injection), and (5) Chronic kidney disease, defined as eGFR <60 mL/min/1.73 m^2^ or creatinine clearance rate <60 mL/min.

### Baseline data collection

The department developed an independent case report form to document patient baseline data and follow-up outcomes. Baseline data were collected by reviewing electronic medical records, while patient grouping and prognosis information were obtained through telephone and outpatient follow-ups and reported in the case report forms. To ensure accuracy, two researchers independently entered the data into SPSS 26.0 software. After entry, the data were reviewed, and patients with more than 5% missing values were excluded. The baseline data parameters for this study were determined based on a review of relevant literature and discussions within the research team. These parameters included general patient information and in-hospital treatment strategies, such as age, sex, body mass index, whether primary PCI was performed, chronic medical history, laboratory test results, culprit artery, and procedural details. The study's primary endpoint was MACCE during follow-up, including cardiac death, myocardial infarction, ischemia-driven revascularization, and stroke. The primary safety endpoint was bleeding events, including major and minor bleeding defined by thrombolysis in myocardial infarction (TIMI). The follow-up period lasted 12 months post-PCI.

### Definitions

In this study, patients initially received DAPT for 12 months following PCI, comprising aspirin 100 mg QD + ticagrelor 60 mg/90 mg BID or aspirin 100 mg QD + clopidogrel 75 mg QD. Only patients who completed this 12-month DAPT period without experiencing major bleeding or MACCE transitioned to SAPT. Based on the SAPT regimen, patients were divided into three groups: 435 patients in the aspirin group (aspirin 100 mg QD), 207 patients in the clopidogrel group (clopidogrel 75 mg QD), and 34 patients in the ticagrelor group (ticagrelor 60 mg/90 mg BID). Due to the small sample size and variable ticagrelor dosing, the ticagrelor group was excluded. SAPT was continued for over 12 months without dose reduction or medication substitution.

In this study, patients in the de-escalation group initially treated with DAPT (aspirin 100 mg QD + ticagrelor 90 mg BID) after PCI had ticagrelor replaced with clopidogrel 75 mg QD or reduced to ticagrelor 60 mg BID at 3 months. DAPT was continued for an additional 9 months. Patients in the clopidogrel group received DAPT (aspirin 100 mg QD + clopidogrel 75 mg QD) for 12 months after PCI. Patients in the ticagrelor group were administered DAPT (aspirin 100 mg QD + ticagrelor 90 mg BID) for 12 months after PCI without any reduction or change in medication.

The primary endpoint was net adverse clinical events (NACE) during the follow-up period, including MACCE and TIMI major bleeding. The secondary endpoints were MACCE and TIMI major bleeding events. MACCE includes composite endpoints of cardiac death, myocardial infarction, revascularization, and stroke, while TIMI major bleeding was defined as fatal hemorrhage, intracranial hemorrhage, or gastrointestinal hemorrhage requiring blood transfusion. TIMI minor bleeding was analyzed, encompassing events such as epistaxis, gingival bleeding, bulbar conjunctival bleeding, skin ecchymosis, hematuria, and fecal occult blood. These events did not require a blood transfusion in clinical practice.

### Follow-up

This study employed a combination of outpatient and telephone follow-ups to monitor patients after PCI. Follow-ups were conducted by a trained data clerk. Outpatient follow-ups were scheduled for 2 weeks, 3 months, 6 months, 12 months, and 24 months post-PCI. For patients who did not attend outpatient visits, follow-up was completed via telephone. The total follow-up duration was 24 months after PCI.

### Statistical analysis

Statistical analysis was performed using SPSS 21.0 software. Measurement data with a normal distribution were expressed as the mean ± standard deviation, and between-group comparisons were conducted using the *t-test*. Non-normally distributed measurement data were presented as the median and interquartile range, with between-group comparisons analyzed using the rank-sum test. Categorical data were expressed as frequencies and percentages, and the chi-square test was used for between-group analysis. Survival rates between the two groups were analyzed using Kaplan–Meier analysis, while Cox multivariate regression analysis was employed to adjust for baseline data. A two-tailed test was applied, and *P* ≤ 0.05 was considered statistically significant.

### Adjudication and outcome validation

Adjudication of outcomes:All clinical outcomes, including MACCE and bleeding events, were independently adjudicated by two cardiologists (M.L. and J.W.) who were blinded to treatment allocation. Disagreements were resolved by consensus or by a third reviewer (Z.X.). Events were confirmed using source documents, including hospital records, discharge summaries, laboratory reports, and imaging studies. Deaths were verified through hospital records or family contact.

### Loss to follow-up and missing data

Loss to follow-up and missing data:Patients who were lost to follow-up before 24 months were censored at the date of last contact. Baseline variables had less than 2% missing values; complete-case analysis was used, as the proportion was negligible.

### Proportional hazards assumption

Proportional hazards assumption:The proportional hazards assumption was tested using Schoenfeld residuals. For all endpoints, the global test and individual covariate tests showed no significant violation (*P* > 0.05), confirming the appropriateness of the Cox models.

## Results

### Baseline data

The analysis results indicated no significant differences between the two groups regarding baseline characteristics such as sex, body mass index, ACS classification, hypertension, type 2 diabetes, cerebrovascular disease, prior myocardial infarction, atrial fibrillation, smoking history, family history of coronary heart disease, or history of revascularization (*P* > 0.05). However, compared to the aspirin group, patients in the clopidogrel group were significantly older (*P* = 0.022). Similarly, the proportion of patients who underwent primary PCI in the clopidogrel group was significantly lower (*P* = 0.010) (Table 1).

**Table 1:** Baseline characteristics.

Characteristic	Aspirin group (*n* = 435)	Clopidogrel group (*n* = 207)	*t*/*χ² value*	*P-value*
Age (year, m ± SD)	62.21 ± 9.38	64.02 ± 9.27	−2.292	0.022
Male (*n*, %)	246 (56.6%)	118 (57.0%)	0.012	0.914
BMI (kg/m^2^, m ± SD)	27.23 ± 20.78	26.19 ± 2.88	0.713	0.476
Presentation				
UAP (*n*, %)	229 (52.6%)	125 (60.4%)	6.937	0.074
NSTEMI (*n*, %)	15 (3.4%)	12 (5.8%)		
STEMI (*n*, %)	189 (43.4%)	69 (33.3%)		
Primary PCI (*n*, %)	167 (38.4%)	58 (28.0%)	6.628	0.010
Medical history (*n*, %)				
Hypertension	295 (67.8%)	146 (70.5%)	0.481	0.488
Type 2 diabetes	156 (35.9%)	69 (33.3%)	0.394	0.530
Cerebrovascular disease	67 (15.4%)	34 (16.4%)	0.111	0.739
OMI	24 (5.5%)	10 (4.8%)	0.132	0.717
Atrial fibrillation	7 (1.1%)	7 (1.1%)	2.066	0.151
Current smoker	176 (40.5%)	90 (43.5%)	0.527	0.468
CAD family history	19 (4.4%)	9 (4.3%)	0.000	0.991
Previous RV	47 (10.8%)	18 (8.7%)	0.686	0.408

SD, standard deviation; BMI, body mass index; UAP, unstable angina pectoris; NSTEMI, non-ST segment elevation myocardial infarction; STEMI, ST segment elevation myocardial infarction; PCI, percutaneous coronary intervention; OMI, old myocardial infarction; CAD, coronary artery disease; RV, revascularization.

### Laboratory test results

The analysis results revealed no statistically significant differences in laboratory test results, including white blood cells, hemoglobin, platelets, fibrinogen, eGFR, uric acid, fasting blood glucose, serum total cholesterol, triglyceride, low-density lipoprotein cholesterol, and high-density lipoprotein cholesterol between the two groups (*P* > 0.05) (Table 2).

**Table 2:** Laboratory tests.

Characteristic	Aspirin group (*n* = 435)	Clopidogrel group (*n* = 207)	*t-value*	*P-value*
WBC (×10^9^/L)	7.45 ± 2.21	7.25 ± 1.84	1.139	0.255
HGB (g/L)	135.82 ± 14.48	135.14 ± 13.77	0.574	0.566
PLT (×10^9^/L)	236.07 ± 51.74	236.74 ± 57.31	0.149	0.881
FIB (g/L)	3.41 ± 0.81	3.45 ± 0.78	0.569	0.569
eGFR	96.97 ± 16.04	94.69 ± 18.85	1.504	0.112
UA (µmol/L)	310.88 ± 82.90	319.43 ± 92.09	1.177	0.240
FBG (mmol/L)	7.20 ± 2.31	7.08 ± 2.28	0.596	0.552
TC (mmol/L)	4.73 ± 2.25	4.75 ± 1.47	0.110	0.912
TG (mmol/L)	2.03 ± 1.01	1.97 ± 0.83	0.733	0.464
LDL-C (mmol/L)	2.76 ± 0.76	2.85 ± 0.80	1.414	0.158
HDL-C (mmol/L)	1.24 ± 0.41	1.13 ± 0.23	0.264	0.792

WBC, white blood cell; HGB, hemoglobin; PLT, platelet; FIB, Fibrinogen; eGFR, estimated glomerular filtration rate; UA, uric acid; FBG, fasting blood glucose; TC, total cholesterol; TG, triglyceride; LDL-C, low-density lipoprotein cholesterol; HDL-C, high-density lipoprotein cholesterol.

### Coronary angiography and interventional procedure

The analysis showed no statistically significant differences between the two groups in terms of culprit vessels, ostial lesions, diffused lesions, chronic total occlusion lesions, in-stent restenosis lesions, number of diseased branches, and number of stents (all *P* > 0.05). However, compared to the aspirin group, the clopidogrel group had a significantly higher incidence of small vessel disease (*P* < 0.001). The incidence of proximal left anterior descending lesions was lower in the clopidogrel group than in the aspirin group (*P* = 0.019), while the rate of complete revascularization was significantly higher in the clopidogrel group (*P* < 0.001) (Table 3).

**Table 3:** Coronary angiography and percutaneous coronary intervention.

Characteristic	Aspirin group (*n* = 435)	Clopidogrel group (*n* = 207)	*t*/*χ² value*	*P-value*
Culprit artery				
LM (*n*, %)	11 (2.5%)	6 (2.9%)	0.074	0.785
LAD (*n*, %)	386 (88.7%)	185 (89.4%)	0.058	0.810
LCX (*n*, %)	299 (68.7%)	141 (68.1%)	0.025	0.874
RCA (*n*, %)	321 (73.8%)	156 (75.4%)	0.181	0.671
Ostial lesion (*n*, %)	111 (25.5%)	53 (25.6%)	0.001	0.981
Diffused lesion (*n*, %)	206 (47.4%)	98 (47.3%)	0.000	0.997
CTO (*n*, %)	14 (3.2%)	5 (2.4%)	0.315	0.575
ISR (*n*, %)	10 (2.3%)	1 (0.5%)	1.774	0.183
Small vessel (*n*, %)	59 (13.6%)	58 (28.0%)	19.668	<0.001
LADp (*n*, %)	126 (29.0%)	42 (20.3%)	5.464	0.019
Complete RV (*n*, %)	122 (28.0%)	104 (50.2%)	30.292	<0.001
Number of diseased arteries (m ± SD)	2.35 ± 0.77	2.36 ± 0.78	0.200	0.841
Number of stents (m ± SD)	1.46 ± 0.63	1.42 ± 0.63	0.879	0.380

LM, left main; LAD, left anterior descending; LCX, left circumflex coronary artery; RCA, right coronary artery; CTO, chronic total occlusion; ISR, in-stent restenosis; LADp, proximal segment of left anterior descending; RV, revascularization; SD, standard deviation.

### Endpoints during follow-up

The results indicated no statistically significant differences between the two groups regarding the incidence of NACE, cardiac death, myocardial infarction, stroke, or bleeding events (including major and minor bleeding) during the follow-up period (*P* > 0.05). However, the clopidogrel group showed a significantly lower incidence of MACCE during follow-up compared to the aspirin group (*P* = 0.031). Additionally, the rate of target vessel revascularization (TVR) was significantly lower in the clopidogrel group during follow-up (*P* = 0.006) (Table 4).

**Table 4:** Endpoints during follow-up.

Characteristic	Aspirin group (*n* = 435)	Clopidogrel group (*n* = 207)	*F/χ² value*	*P*-value
NACE (*n*, %)	45 (10.3%)	13 (6.3%)	2.820	0.093
MACCE (*n*, %)	40 (9.2%)	9 (4.3%)	4.675	0.031
Cardiac death (*n*, %)	1 (0.2%)	4 (1.9%)	3.288	0.070
MI (*n*, %)	7 (1.6%)	2 (1.0%)	0.083	0.773
TVR (*n*, %)	28 (6.4%)	3 (1.4%)	7.592	0.006
Stroke (*n*, %)	9 (2.1%)	1 (0.5%)	1.382	0.240
Bleeding event (*n*, %)	48 (11.0%)	21 (10.1%)	0.116	0.734
Major bleeding	9 (2.1%)	5 (2.4%)	0.078	0.781
Minor bleeding	42 (9.7%)	19 (9.2%)	0.037	0.847

NACE, net adverse clinical event; MACCE, major adverse cardiovascular and cerebrovascular event; MI, myocardial infarction; TVR, target vessel revascularization.

### Kaplan–Meier survival curve

**Figure 2:**
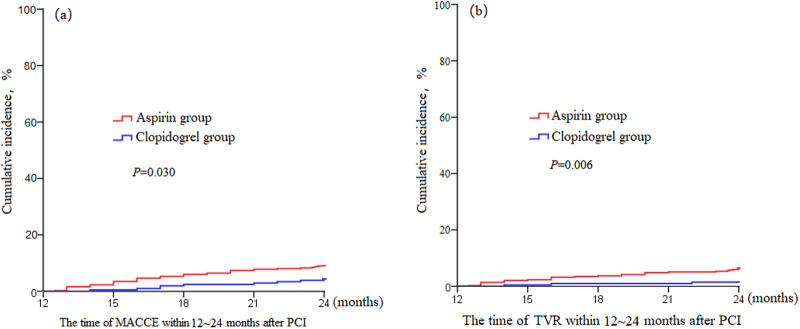
Kaplan–Meier survival curve of incidence MACCE and TVR 12–24 months after percutaneous coronary intervention. **(a)** MACCE cumulative incidence. **(b)** TVR cumulative incidence. MACCE, major adverse cardiovascular and cerebrovascular event; TVR, target vessel revascularization.

The Kaplan–Meier survival curve demonstrated that the cumulative incidence of MACCE was significantly lower in the clopidogrel group compared to the aspirin group (*P* = 0.030). Similarly, the cumulative incidence of TVR was lower in the clopidogrel group (*P* = 0.006) (Figure 2a, b).

### COX regression results of the prognosis of patients

Cox regression analysis was performed using variables with baseline differences and other relevant influencing factors as independent variables. The results showed that the incidence of MACCE and TVR in the clopidogrel group were 0.458 times [*hazard ratio [HR]:* 0.458; *95% confidence interval [CI]:* 0.222–0.944; *P* = 0.034] and 0.220 times (*HR:* 0.220; *95% CI:* 0.067–0.724: *P* = 0.013) that of the aspirin group, respectively. However, the Cox regression analysis of differential indicators such as age, primary PCI, small vessel disease, proximal left anterior descending lesions, and complete revascularization showed no statistically significant results (all *P* > 0.05) (Table 5).

**Table 5:** Cox regression.

Endpoints	Variables	Quotient		*HR*	95% CI	*P-value*
		*B*	*SE*			
MACCE	Clopidogrel group (Reference: aspirin group)	−0.780	0.369	0.458	0.222–0.944	0.034
TVR	Clopidogrel group (Reference: aspirin group)	−1.514	0.607	0.220	0.067–0.724	0.013

MACCE, major adverse cardiovascular and cerebrovascular event; TVR, target vessel revascularization; CI, confidence interval; HR, hazard ratio.

### Loss to follow-up

Follow-up and missing data: Of the 642 patients initially included, 17 (2.6%) were lost to follow-up before 24 months [9 (2.1%) in the aspirin group and 8 (3.9%) in the clopidogrel group]. The loss-to-follow-up rate was similar between groups (*P* = 0.20).

### Medication adherence and cross-over

Medication adherence and cross-over:Adherence to SAPT (defined as taking the prescribed medication for ≥80% of the time without interruption >7 days) was 93.8% in the aspirin group and 94.2% in the clopidogrel group. Cross-over (switching to the other SAPT or adding a second antiplatelet agent) occurred in 12 patients (2.8%) in the aspirin group and 7 patients (3.4%) in the clopidogrel group. All patients were analyzed according to the intention-to-treat principle based on their original group assignment.

### Bleeding outcomes HR

Bleeding outcomes:Cox regression analysis showed no significant difference in major bleeding between the two groups (HR: 0.92, 95% CI: 0.31–2.73, *P* = 0.88), confirming that clopidogrel SAPT did not increase the risk of major bleeding compared to aspirin.

## Discussion

This single-center cohort study, using real-world data, retrospectively analyzed 642 dual high-risk patients with ACS who underwent PCI. After 12 months of DAPT, these patients transitioned to SAPT (aspirin or clopidogrel) for more than 12 months, with the study observing the impact of the two SAPT regimens on patient outcomes. The findings indicated that, compared to aspirin, clopidogrel as a SAPT regimen after 12 months of DAPT was associated with a lower incidence of MACCE and TVR in patients with ACS at dual high-risk of ischemia and bleeding after PCI, without increasing the risk of bleeding.

Traditionally, clinical practice has favored discontinuing P2Y_12_ receptor inhibitors and selecting aspirin for SAPT (13). However, studies have shown that aspirin can increase the risk of gastrointestinal bleeding by 2–4 times (14). Consequently, recent studies have explored the safety and efficacy of P2Y_12_ receptor inhibitors as SAPT for secondary prevention in patients with CAD following DAPT post-PCI. For instance, Taek et al. studied patients who received drug-eluting stents and DAPT for 12 months, dividing them into aspirin and clopidogrel groups. After 36 months of SAPT, clopidogrel was shown to reduce the incidence of cardiac death and myocardial infarction (15). Similarly, Sun et al. compared the efficacy of aspirin and clopidogrel as SAPT in 1,819 patients with acute myocardial infarction who underwent PCI. After 12 months of DAPT, 534 asymptomatic patients switched to clopidogrel monotherapy, and 1,285 switched to aspirin monotherapy. Their findings revealed comparable incidence of NACE between the two groups, with no statistically significant difference in the incidence of major bleeding events and MACCE (16). Additionally, a meta-analysis by Jun et al. on the efficacy of aspirin and clopidogrel in patients with stable coronary heart disease found no statistically significant differences in all-cause mortality, myocardial infarction, stroke, or bleeding [Bleeding Academic Research Consortium (BARC) grade 3 and above] between clopidogrel and aspirin monotherapy groups (17). Therefore, long-term SAPT drug selection in patients undergoing PCI remains controversial, particularly for dual high-risk individuals, and no previous studies have specifically addressed this subgroup. This study seeks to fill that gap and provide insight into SAPT drug selection for these patients.

Currently, clinical evidence on antiplatelet regimens for dual high-risk individuals after 12 months of DAPT post-PCI is limited, with no clear recommendation in the guidelines. The 2020 ESC guidelines for non-ST-elevation acute coronary syndrome (NSTE-ACS) suggest evaluating the bleeding risk of patients with NSTE-ACS post-PCI to determine antiplatelet therapy: aspirin monotherapy is recommended for high bleeding risk patients after 3 months of DAPT, while clopidogrel monotherapy is proposed for extremely high bleeding risk patients after 1 month of DAPT; for low bleeding risk patients, further assessment of ischemic risk is advised to guide more precise antiplatelet strategies (18). However, this model does not address antiplatelet regimens for patients with dual high-risk, highlighting a lack of evidence in this area of the guidelines. Previous studies have demonstrated that, compared to aspirin alone, extending DAPT beyond 12 months can significantly reduce MACCE but also increase the risk of bleeding (7–9). Therefore, prolonging DAPT for more than 12 months may not be suitable for patients with ACS at dual high-risk for ischemia and bleeding. In this study, we enrolled patients with dual high-risk and transitioned them to two different SAPT strategies after completing the standard 12-month DAPT regimen. The follow-up period lasted up to 24 months after PCI. The study aimed to evaluate the SAPT regimens for patients with dual high-risk during the 12–24 month period post-PCI, as the optimal DAPT duration for these patients has not been comprehensively explored.

In our study, approximately 59.9% of the ACS population exhibited dual high-risk characteristics, which are often prevalent in real-world settings. However, this group has historically received limited attention. Although multiple scoring systems exist for scoring ischemia and bleeding risks in patients with ACS after PCI, each has inherent limitations. The 2023 ESC guidelines for ACS management proposed a high ischemia score standard but introduced the subjective concept of “complex coronary heart disease,” which relies on clinical judgment. Moreover, these standards are applicable only to patients with NSTE-ACS (19). Similarly, the GRACE ischemic score applies to NSTE-ACS, providing guidance on PCI timing and predicting the risk of death within 6 months of discharge; however, it does not evaluate prognosis 12–24 months post-PCI (20). To evaluate DAPT duration beyond 12 months post-PCI, multiple guidelines recommend the DAPT scoring system, but it omits clinical factors such as systemic inflammatory diseases, renal dysfunction, and complex surgical factors, limiting its ability to fully assess ischemic risk (21). The CRUSADE score is effective for predicting in-hospital bleeding risk but does not account for patient comorbidities or past bleeding history, reducing its applicability for assessing bleeding risk post-hospitalization (22). In contrast, the OPT-BIRISK evaluation system used in this study is more suitable for the Chinese ACS population. Unlike other scoring methods, OPT-BIRISK incorporates PCI-specific factors and assesses ischemia and bleeding risks, allowing for a more comprehensive evaluation of dual high-risk patients with ACS undergoing PCI (10). In addition, the “East Asia paradox”, which refers to ethnic differences in clopidogrel response (23), further supports the use of OPT-BIRISK for this predominantly Chinese study population. This study assessed high ischemic and bleeding risks during hospitalization but did not conduct dynamic evaluations 12 months post-PCI. However, the OPT-BIRISK study includes indicators such as glomerular filtration rate and creatinine, which can improve with standardized treatment and potentially alter dual high-risk assessments over time. In this study, dynamic evaluation was not performed primarily because the proportion of patients with baseline eGFR <60 mL/min/1.73 m^2^ or creatinine clearance rate <60 mL/min was very low. Therefore, these factors had minimal impact on the final evaluation outcomes, and the likelihood of significant improvement in other scoring indicators was also low.

The results of this study demonstrated that SAPT with clopidogrel effectively reduces MACCE and TVR without increasing major bleeding risk. Clopidogrel is more effective than aspirin at inhibiting adenosine 5′-diphosphate-mediated platelet aggregation and activation (24). A randomized controlled trial was conducted to investigate the efficacy of transitioning from DAPT to aspirin or clopidogrel monotherapy in patients post-PCI. A total of 5,438 patients with no clinical events after 6–18 months of DAPT post-PCI were randomly assigned to the aspirin monotherapy group (2,728 cases) or the clopidogrel monotherapy group (2,710 cases) (25). After 24 months of follow-up, the clopidogrel group showed significantly lower rates of all-cause mortality, ACS readmissions, and bleeding risk (BARC ≥3) compared to the aspirin group, indicating the superiority of clopidogrel for long-term SAPT after PCI. These findings are consistent with the results of this current study, with the key distinction being that our study focused specifically on dual high-risk ACS populations. This population, because of their multiple risk factors, often requires a more precise balance between ischemia and bleeding risks. However, the conclusions of the OPT-PEACE study differ from ours (26). The OPT-PEACE study was the first to use capsule endoscopy to assess the gastrointestinal mucosal damage caused by different antiplatelet regimens in patients who underwent PCI. After 6 months of DAPT post-PCI, patients were randomized into three groups (aspirin + clopidogrel, aspirin + placebo, or clopidogrel + placebo) and underwent capsule endoscopy at baseline, 6 months, and 12 months after PCI. The study found that SAPT reduced the incidence of gastrointestinal injury, mild bleeding events, and gastrointestinal bleeding compared to 12-month DAPT. However, clopidogrel and aspirin as SAPT exhibited comparable degrees of gastrointestinal mucosal damage. Various factors may explain these differences. First, the OPT-PEACE study included patients without bleeding as detected by capsule endoscopy, whereas our study focused on a population with higher bleeding risk. Second, the DAPT duration in the OPT-PEACE study was 6 months, compared to 12 months in our study. Prolonged antiplatelet therapy may increase the risk of gastrointestinal damage, making differences more apparent in the long-term follow-up compared to short-term observations. In addition, this study considered major bleeding events beyond gastrointestinal bleeding, including cerebral hemorrhage. In recent years, there has been debate over which SAPT regimen, aspirin or clopidogrel, is more beneficial in reducing gastrointestinal bleeding. While aspirin can cause gastric mucosal damage through various mechanisms, clopidogrel offers relative advantages in this regard. However, studies have shown that clopidogrel can delay the healing of gastric ulcers (27). Additionally, proton pump inhibitors (PPIs), commonly used by patients with gastrointestinal conditions, can interfere with clopidogrel metabolism, reducing the production of its active metabolites and potentially diminishing its antithrombotic effect. This has also triggered important clinical considerations. For patients with a history of gastrointestinal diseases requiring PPIs, the antithrombotic effect of clopidogrel may be compromised, and it may further delay gastric ulcer healing. In such cases, clopidogrel has shown certain disadvantages in terms of balancing antithrombotic efficacy and bleeding prevention. Therefore, exploring the benefits of different SAPT regimens in gastrointestinal bleeding should focus on factors such as prior gastrointestinal diseases, PPI use, and the duration of DAPT. The OPT-BIRISK study, which focused on dual high-risk populations, compared DAPT with clopidogrel monotherapy (10). The findings showed that SAPT with clopidogrel was superior in reducing clinically relevant bleeding and MACCE. However, the incidence of major bleeding events was relatively low in this study. This may be attributed to two factors: (1) major bleeding events occurred 12–24 months after PCI, during which patients transitioned from DAPT, associated with higher bleeding risk, to SAPT; and (2) patients who experienced MACCE or major bleeding within the first 12 months were excluded, potentially eliminating some very high-risk individuals. These findings also highlight the need for future research to develop more comprehensive scoring systems that incorporate a broader range of risk factors. Such systems should aim to differentiate “very high-risk” patients from those at high-risk of ischemia and bleeding to better inform treatment strategies.

The baseline data in this study revealed that the clopidogrel group had a higher average age, a higher proportion of small vessel lesions and complete revascularization, and a lower proportion of primary PCI and proximal left anterior descending lesions compared to the aspirin group. The higher average age in the clopidogrel group suggests a certain subjective preference by researchers in prescribing SAPT to older patients or those with a higher risk of bleeding. Furthermore, differences in coronary artery lesion characteristics and the PCI procedure between the two groups may influence the study outcomes and directly affect patient outcomes. As a cohort study, a key limitation is the lack of randomization, which can introduce baseline imbalance. To address this, Cox regression analysis was employed to adjust for baseline differences and minimize their impact on the study results.

Regarding research design, in this study, patients were divided into aspirin and clopidogrel groups based on their transition from DAPT to SAPT at 12 months post-PCI. On the one hand, the study did not restrict the choice of DAPT regimens during the initial 12 months after PCI. Patients receiving DAPT could use aspirin in combination with clopidogrel or ticagrelor as P2Y_12_ receptor inhibitors. Different types and doses of P2Y_12_ receptor inhibitors can impact patient prognoses. However, patients who experienced MACCE or major bleeding events during DAPT were excluded during the screening. In contrast, the proportion of individuals who chose ticagrelor as their SAPT regimen was relatively low, and the dosages used varied. Consequently, these patients were not included in the study. The PLATO trial demonstrated that the incidence of fatal intracranial hemorrhage in the ticagrelor group was 10 times that of the clopidogrel group (2). Although ticagrelor is associated with a higher incidence of major bleeding, it offers significant advantages over clopidogrel in specific cases, such as those with platelet hyperreactivity or interactions with PPIs. Ticagrelor is available in two doses (90 mg and 60 mg), which can impact outcomes in certain patient populations. In clinical practice, platelet function testing and CYP2C19 genetic testing can further refine antiplatelet therapy strategies when choosing between clopidogrel and ticagrelor. Platelet function testing directly reflects platelet reactivity, while CYP2C19 genetic testing identifies metabolic limitations that may explain platelet hyperreactivity (28). These tests are also valuable for guiding escalation or de-escalation of therapy in patients with high or low platelet reactivity. The TAILOR-PCI study found that using CYP2C19 genetic testing to guide the selection of oral P2Y_12_ receptor inhibitors in patients undergoing PCI did not significantly improve overall prognosis compared to clopidogrel. However, a detailed analysis of the first 3 months revealed that ticagrelor was significantly more effective than clopidogrel in CYP2C19*2/*3 carriers identified through genetic testing (29). Therefore, combining dual high-risk bleeding scores with platelet function testing and CYP2C19 genetic testing to explore precise antiplatelet therapy for dual high-risk patients could become a future research direction.

In summary, although the OPT-BIRISK score is the most appropriate scoring system for this study, it has limitations in its definition of high bleeding risk factors, such as the exclusion of prior bleeding history. Furthermore, it does not offer detailed guidance on selecting SAPT after 12 months post-PCI. Future research should focus on designing a more comprehensive scoring system that incorporates additional relevant indicators to improve predictive performance and provide more effective treatment recommendations.It should be acknowledged that the initial DAPT regimen during the first 12 months after PCI varied among patients (aspirin + clopidogrel or aspirin + ticagrelor), which may have residual confounding effects on the observed outcomes. However, all patients who completed 12 months of DAPT without MACCE or major bleeding were included regardless of their initial P2Y12 inhibitor, and the primary focus of this study was the comparison of SAPT regimens after this period. Due to the modest sample size, we were unable to perform stratified analyses by initial DAPT type; future larger studies are needed to explore this question.

## Conclusion

For patients with ACS at dual high-risk of ischemia and bleeding, transitioning to clopidogrel as the SAPT regimen after PCI and 12 months of DAPT reduces the incidence of MACCE and TVR compared to aspirin without increasing the risk of major bleeding events.

## Limitations

This study has some limitations, including the following: (1) It is a retrospective cohort study with a small sample size and a low incidence of primary endpoints, which may reduce its statistical power to some extent. (2) The choice of antiplatelet drugs was subjective, with no randomization of patients into groups, potentially leading to heterogeneity between the two groups. However, to mitigate this bias, a detailed analysis of baseline data was performed, and Cox regression analysis was used to adjust for baseline differences. (3) Before initiating DAPT, doctors conducted a preliminary “pre-evaluation” of ischemic and bleeding risks based on clinical indicators, medical history, and PCI conditions, leading to the subjective selection of P2Y_12_ receptor inhibitors. The impact of different DAPT regimens within the first 12 months requires further investigation. The real-world subjectivity in medication selection could also contribute to biased results. (4) The small number of patients treated with ticagrelor as an SAPT regimen limited the study to include only aspirin and clopidogrel. Future studies should include ticagrelor to explore the impact of various SAPT regimens on the prognosis of dual high-risk patients. (5) Although the scoring system for ischemia and bleeding risks used in this study was based on the OPT-BIRISK definition, which is the most suitable for this study, it does not fully capture ischemic and bleeding risks. Additionally, the dual high-risk assessment of patients in this study was conducted during hospitalization, without re-evaluation after 12 months of DAPT. These limitations highlight areas for further research to strengthen the evidence base for SAPT regimens in dual high-risk ACS populations.due to the retrospective nature of this study, we were unable to adjust for certain important confounders related to bleeding risk, such as proton pump inhibitor use and prior bleeding history, as these variables were not consistently recorded in the electronic medical records. Although the OPT-BIRISK criteria partially capture bleeding risk through factors such as female sex, diabetes, CKD, and prior stroke, residual confounding may still exist. Future prospective studies should systematically collect these variables to enable more comprehensive adjustment.while propensity score matching or weighting could provide additional robustness in confounding control, these methods were not employed due to the modest sample size and the risk of overfitting. Multivariable Cox regression was used as the primary method for adjustment, including all clinically relevant variables and those with baseline imbalance. Nevertheless, residual confounding may still influence the findings.genetic testing for CYP2C19 loss-of-function alleles or platelet function testing (e.g., Multiplate) was not routinely performed during the study period. Therefore, we cannot assess whether clopidogrel's effectiveness was influenced by genetic variants or on-treatment platelet reactivity. Future studies incorporating pharmacogenomic or platelet function testing may help personalize SAPT selection in dual high-risk patients.

The significantly higher rate of complete revascularization in the clopidogrel group represents an important confounder for the observed reduction in TVR. Although we adjusted for complete revascularization in the Cox regression model, the large effect size (HR: 0.220) raises concerns about residual confounding and may not be entirely attributable to clopidogrel therapy. Therefore, the TVR finding should be interpreted with caution and considered hypothesis-generating. The primary conclusion of this study is based on the MACCE endpoint, which remained significant after adjustment.The baseline imbalances between the two groups (age, lesion characteristics, revascularization status) reflect real-world physician preference and introduce potential selection bias. Although we adjusted for these variables in multivariable Cox regression, residual confounding may still influence the findings. Propensity score matching was not performed due to the modest sample size and low event rates, which would limit the feasibility and statistical power of such an analysis.The low number of events, particularly in the clopidogrel group (e.g., only 3 TVR events), increases the risk of overfitting in the multivariable Cox regression models. Although we limited the number of covariates to those with baseline imbalance, the ratio of events to variables was below the recommended 10:1 for some endpoints. Therefore, the adjusted hazard ratios, especially for TVR, should be interpreted with caution and considered hypothesis-generating. Larger prospective studies are needed to confirm these findings.

## Data Availability

The original contributions presented in the study are included in the article/supplementary material, further inquiries can be directed to the corresponding author.

## References

[1] Yusuf S Zhao F Mehta SR Chrolavicius S Tognoni G Fox KK Clopidogrel in unstable angina to prevent recurrent events trial investigators. Effects of clopidogrel in addition to aspirin in patients with acute coronary syndromes without ST-segment elevation. N Engl J Med. (2001) 345(7):494–502. 10.1056/NEJMoa010746 11519503

[2] Wallentin L Becker RC Budaj A Cannon CP Emanuelsson H Held C Ticagrelor versus clopidogrel in patients with acute coronary syndromes. N Engl J Med. (2009) 361(11):1045–57. 10.1056/NEJMoa0904327 19717846

[3] Mehta SR Yusuf S Peters RJ Bertrand ME Lewis BS Natarajan MK Effects of pretreatment with clopidogrel and aspirin followed by long-term therapy in patients undergoing percutaneous coronary intervention: the PCI-CURE study. Lancet. (2001) 358(9281):527–33. 10.1016/s0140-6736(01)05701-4 11520521

[4] Wiviott SD Braunwald E McCabe CH Montalescot G Ruzyllo W Gottlieb S Prasugrel versus clopidogrel in patients with acute coronary syndromes. N Engl J Med. (2007) 357:2001–15. 10.1056/NEJMoa0706482 17982182

[5] Valgimigli M Bueno H Byrne RA Collet JP Costa F Jeppsson A 2017 ESC focused update on dual antiplatelet therapy in coronary artery disease developed in collaboration with EACTS: the task force for dual antiplatelet therapy in coronary artery disease of the European Society of Cardiology (ESC) and of the European association for cardio-thoracic surgery (EACTS). Eur Heart J. (2018) 39(3):213–60. 10.1093/eurheartj/ehx419 28886622

[6] Levine GN Bates ER Bittl JA Brindis RG Fihn SD Fleisher LA 2016 ACC/AHA guideline focused update on duration of dual antiplatelet therapy in patients with coronary artery disease: a report of the American College of Cardiology/American Heart Association task force on clinical practice guidelines. J Am Coll Cardiol. (2016) 68(10):1082–115. 10.1016/j.jacc.2016.03.513 27036918

[7] Mauri L Kereiakes DJ Yeh RW Driscoll-Shempp P Cutlip DE Steg PG DAPT Study investigators. Twelve or 30 months of dual antiplatelet therapy after drug-eluting stents. N Engl J Med. (2014) 371(23):2155–66. 10.1056/NEJMoa1409312 25399658 PMC4481318

[8] Bonaca MP Bhatt DL Cohen M Steg PG Storey RF Jensen EC PEGASUS-TIMI 54 steering committee and investigators. Long-term use of ticagrelor in patients with prior myocardial infarction. N Engl J Med. (2015) 372(19):1791–800. 10.1056/NEJMoa1500857 25773268

[9] Verdoia M Khedi E Ceccon C Suryapranata H De Luca G. Duration of dual antiplatelet therapy and outcome in patients with acute coronary syndrome undergoing percutaneous revascularization: a meta-analysis of 11 randomized trials. Int J Cardiol. (2018) 264:30–8. 10.1016/j.ijcard.2018.02.095. Erratum in: Int J Cardiol. 2018 November 15;271:407. doi: 10.1016/j.ijcard.2018.06.016 29776573

[10] Li Y Jing Q Wang B Wang X Li J Qiao S Extended antiplatelet therapy with clopidogrel alone versus clopidogrel plus aspirin after completion of 9- to 12-month dual antiplatelet therapy for acute coronary syndrome patients with both high bleeding and ischemic risk. Rationale and design of the OPT-BIRISK double-blinded, placebo-controlled randomized trial. Am Heart J. (2020) 228:1–7. 10.1016/j.ahj.2020.07.005 32739652 PMC7346838

[11] Han Y Chen J Qiu M Li Y Li J Feng Y Predicting long-term ischemic events using routine clinical parameters in patients with coronary artery disease: the OPT-CAD risk score. Cardiovasc Ther. (2018) 36(5):e12441. 10.1111/1755-5922.12441 29869835

[12] ESC Scientific Document Group. 2017 ESC guidelines for the management of acute myocardial infarction in patients presenting with ST-segment elevation: the task force for the management of acute myocardial infarction in patients presenting with ST-segment elevation of the European Society of Cardiology (ESC). Eur Heart J. (2018) 39(2):119–77. 10.1093/eurheartj/ehx393 28886621

[13] Hong SJ Shin DH Kim JS Kim BK Ko YG Choi D IVUS-XPL Investigators. 6-month versus 12-month dual-antiplatelet therapy following long everolimus-eluting stent implantation: the IVUS-XPL randomized clinical trial. JACC Cardiovasc Interv. (2016) 9(14):1438–46. 10.1016/j.jcin.2016.04.036 27212028

[14] Lanas A García-Rodríguez LA Arroyo MT Gomollón F Feu F González-Pérez A Asociación española de gastroenterología. Risk of upper gastrointestinal ulcer bleeding associated with selective cyclo-oxygenase-2 inhibitors, traditional non-aspirin non-steroidal anti-inflammatory drugs, aspirin and combinations. Gut. (2006) 55(12):1731–8. 10.1136/gut.2005.080754 16687434 PMC1856452

[15] Park TK Song YB Ahn J Carriere KC Hahn JY Yang JH Clopidogrel versus aspirin as an antiplatelet monotherapy after 12-month dual-antiplatelet therapy in the era of drug-eluting stents. Circ Cardiovasc Interv. (2016) 9(1):e002816. 10.1161/CIRCINTERVENTIONS.115.002816 26755571

[16] Sim DS Jeong MH Kim HS Gwon HC Seung KB Rha SW KAMIR-NIH registry investigators. Clopidogrel versus aspirin after dual antiplatelet therapy in acute myocardial infarction patients undergoing drug-eluting stenting. Korean Circ J. (2020) 50(2):120–9. 10.4070/kcj.2019.0166 31845550 PMC6974667

[17] Yuan J Xu GM Ding J. Aspirin versus clopidogrel monotherapy for the treatment of patients with stable coronary artery disease: a systematic review and meta-analysis. Adv Ther. (2019) 36(8):2062–71. 10.1007/s12325-019-01004-6 31154631 PMC6822863

[18] Collet JP Thiele H Barbato E Barthélémy O Bauersachs J Bhatt DL 2020 ESC guidelines for the management of acute coronary syndromes in patients presenting without persistent ST-segment elevation. Eur Heart J. (2021) 42(14):1289–367. 10.1093/eurheartj/ehaa575 32860058

[19] Han Y Chen J Qiu M Li Y Li J Feng Y Predicting long-term ischemic events using routine clinical parameters in patients with coronary artery disease: the OPT-CAD risk score. Cardiovasc Ther. (2018) 36:e12441. 10.1111/1755-5922.12441 29869835

[20] Urban P Mehran R Colleran R Angiolillo DJ Byrne RA Capodanno D Defining high bleeding risk in patients undergoing percutaneous coronary intervention: a consensus document from the academic research consortium for high bleeding risk. Eur Heart J. (2019) 40(31):2632–53. 10.1093/eurheartj/ehz372 31116395 PMC6736433

[21] Huo Y Jeong YH Gong Y Wang D He B Chen J 2018 Update of expert consensus statement on antiplatelet therapy in east Asian patients with ACS or undergoing PCI. Sci Bull. (2019) 64(03):166–79. 10.1016/j.scib.2018.12.020. 36659616

[22] Abu-Assi E Raposeiras-Roubin S Lear P Cabanas-Grandío P Girondo M Rodríguez-Cordero M Comparing the predictive validity of three contemporary bleeding risk scores in acute coronary syndrome. Eur Heart J Acute Cardiovasc Care. (2012) 1(3):222–31. 10.1177/2048872612453924 24062910 PMC3760544

[23] Kim HK Tantry US Smith SC Jr Jeong MH Park SJ Kim MH The east Asian paradox: an updated position statement on the challenges to the current antithrombotic strategy in patients with cardiovascular disease. Thromb Haemost. (2021) 121(4):422–32. 10.1055/s-0040-1718729 33171520

[24] Moshfegh K Redondo M Julmy F Wuillemin WA Gebauer MU Haeberli A Antiplatelet effects of clopidogrel compared with aspirin after myocardial infarction: enhanced inhibitory effects of combination therapy. J Am Coll Cardiol. (2000) 36(3):699–705. 10.1016/s0735-1097(00)00817-2 10987587

[25] Koo BK Kang J Park KW Rhee TM Yang HM Won KB HOST-EXAM investigators. Aspirin versus clopidogrel for chronic maintenance monotherapy after percutaneous coronary intervention (HOST-EXAM): an investigator-initiated, prospective, randomised, open-label, multicentre trial. Lancet. (2021) 397(10293):2487–96. 10.1016/S0140-6736(21)01063-1 34010616

[26] Han Y Liao Z Li Y Zhao X Ma S Bao D Magnetically controlled capsule endoscopy for assessment of antiplatelet therapy-induced gastrointestinal injury. J Am Coll Cardiol. (2022) 79(2):116–28. 10.1016/j.jacc.2021.10.028 34752902

[27] Luo JC Peng YL Chen TS Huo TI Hou MC Huang HC Clopidogrel inhibits angiogenesis of gastric ulcer healing via downregulation of vascular endothelial growth factor receptor 2. J Formos Med Assoc. (2016 Sep) 115(9):764–72. 10.1016/j.jfma.2015.07.022 26315480

[28] Campo G Valgimigli M Gemmati D Percoco G Catozzi L Frangione A Poor responsiveness to clopidogrel: drug-specific or class-effect mechanism? Evidence from a clopidogrel-to-ticlopidine crossover study. J Am Coll Cardiol. (2007) 50(12):1132–7. 10.1016/j.jacc.2007.04.092 17868803

[29] Pereira NL Farkouh ME So D Lennon R Geller N Mathew V Effect of genotype-guided oral P2Y12 inhibitor selection vs conventional clopidogrel therapy on ischemic outcomes after percutaneous coronary intervention: the TAILOR-PCI randomized clinical trial. JAMA. (2020) 324(8):761–71. 10.1001/jama.2020.12443 32840598 PMC7448831

